# Synthesis of Symmetrical and Non-symmetrical Diimines from Dimedone

**DOI:** 10.3390/molecules14062278

**Published:** 2009-06-24

**Authors:** Bahjat A. Saeed, Ibrahim A. Musad

**Affiliations:** University of Basrah, College of Education, Chemistry Department, Basrah, Iraq

**Keywords:** dimedone, diimines, enaminothiones, B3LYP/6-31G(d,p)

## Abstract

Symmetrical and non-symmetrical diimines derived from dimedone were synthesized by the reaction of their corresponding enaminothiones with primary amines. The synthesized compounds were characterized using micro analytical data and NMR spectroscopy. Theoretical calculations by B3LYP/6-31G(d,p) level of theory show that the enolic form is the most stable within the possible tautomeric forms of the compounds.

## Introduction

Diimines of β-diketones have received significantly increasing attention which stems from the scope for variation of the substituents on the nitrogen atoms [1]. In addition, they have been used as precursors to some heterocyclic compounds such as imidazolines, pyrimidines and carbapenem intermediates [2,3]. There are two synthetic procedures for the conversion of β-diketone into a β-diketimine [1]. The first is to convert the diketone to the dioxyalkanone, which is then reacted with an excess amount of the amine. The second one is a diacetal reaction with the amine hydrochloride. Another synthetic but indirect route is the addition of ketamines to nitriles. Lee *et al*. [4] prepared the trialkyldiketamines by reaction of aromatic nitriles and Grignard reagent. Qian *et al*. [5] used acetylacetone monoimine to prepare the corresponding diimines by the reaction with the hydrochloride salt of the amine. In one method dibenzoylmethane was reacted with a large excess of aniline and benzylamine in presence of titanium(IV) chloride to give the corresponding diimine [3].

A general feature of those methods is the limited flexibility for varying substituents and the yield of only symmetrical diimines with identical substitution on both imino groups. The thione group has much better reactivity than the carbonyl group with amines to give monoketamines [6] and thiodibenzoylmethane was used to prepare the corresponding ketamines through relatively fast and clean reaction with amines. In this work we report about the synthesis of both symmetrical and non-symmetrical diimines of dimedone by the reaction of enaminothiones and primary amines.

## Results and Discussion

The diimines 1-6 were synthesized by the reaction of enaminothiones and primary amines by refluxing in chloroform. The reaction was accompanied by the evolution of hydrogen sulphide, indicating that it occurred at the thione group with the replacement of sulphur with nitrogen. Moderate yields ranging from 38 to 62% were obtained for the diimines.

**Scheme 1:**
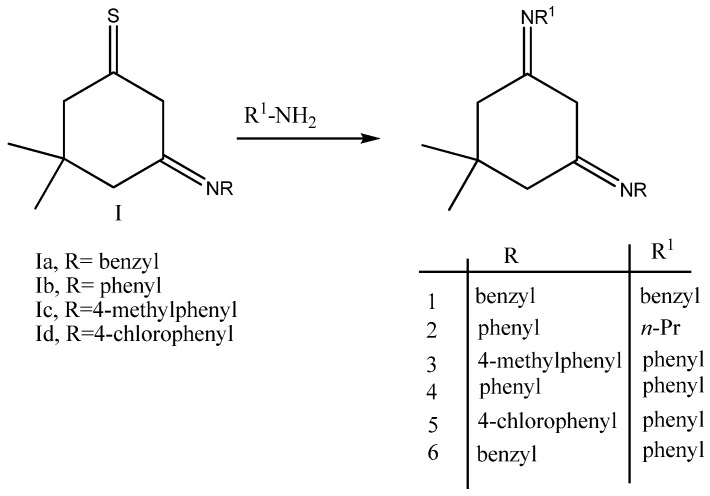
Schematic route for the preparation of the diimines.

**Figure 1:**
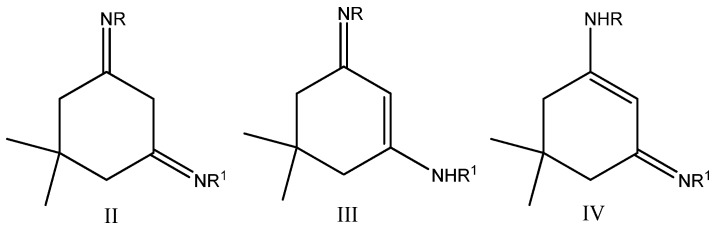
The possible tautomers of the diimines derived from dimedone.

Generally, the compounds can be present as a tautomeric mixture of the forms II, III, or IV (Figure 1). The ^1^H-NMR data of these diimines (as shown in the Experimental) is characterized by two main signals within the ranges 4.86-5.81 and 10.29-11.50 ppm. These ^1^H-NMR signals can be attributed to the C*H*=C proton (4.86-5.81 ppm) and to the NH proton (10.29-11.50 ppm) in the enolic forms III or IV. This is in agreement with the case of enaminones (isoelectronic analogues of the diimines) which on the basis of NMR spectra [7,8] and theoretical study [9] are characterized by the enolic tautomer [10]. For tautomer II to be present the spectra must contain a corresponding signal for the N=C–C*H*_2_–C=N protons. The absence of such signal from the spectra excludes the presence of this tautomer.

The spectra also showed that while the symmetrical molecules (R= R^1^) have only one signal for the NH proton, the asymmetrical molecules (R≠ R^1^) have two signals within the same range. This suggests a tautomeric equilibrium of two enolic species in the later molecules in which the NH protons adapt two different chemical environments (III and IV). This case could not be the result of more than one conformers as indicated by the presence of only one singlet for the NH proton in the symmetrical molecules. The presence of only one NH proton peak in spectra of the non-symmetrical compounds in DMSO may be attributed to the presence of only one enolic tautomer in this solvent.

**Figure 2:**
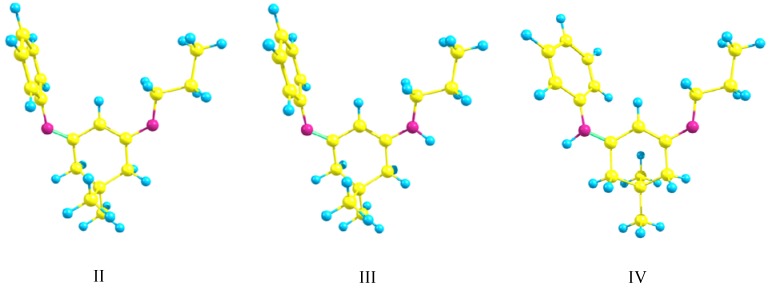
DFT-calculated optimized structures of the possible tautomers for the diimine with R = *n*-Pr and R^1^ = Ph using B3LYP/6-31G(d,p) level of theory.

To shed some light on this case the possible tautomers of compound **2** werere studied theoretically at DFT level[11] using B3LYP/6-31G(d,p) level of theory. Figure 2 shows the calculated structures of the proposed tautomers.

From Table 1 it could be seen that the calculated energy values indicate that the two enolic forms III and IV have almost similar energies and thus comparable stabilities, while the form II has much more energy and accordingly less stability in comparison with the other tautomers. This may be rationalized on the basis that the enolic forms have a conjugation through the N=C–C=CH–NH system and this conjugation is responsible for the stabilization energy obtained by the two enolic forms. The conjugation, visible in the nearly planar end of the central six-membered ring, is also reproduced from calculation. The calculated energies show also that within the enolic forms the most stable structure is that in which the aromatic substituent on the nitrogen atom is attached to the imino rather than the amino group.

**Table 1:** Calculated total electronic energies (a.u.) of the three tautomers.

	Method	B3LYP/6-31G(d,p)	B3LYP/6-31G(d)	B3WP91/6-31G(d)	HF/6-31G(d)
Tautomer					
II		–687.530	-696.301	-696.242	-692.427
III		–696.631	-696.310	-696.249	-692.435
IV		–696.775	-687.395	-688.313	-684.111

Vibrational spectroscopy is used in organic chemistry for the identification of functional groups of organic compounds. Assignments for the complex systems can be proposed on the basis of frequency agreement between the computed harmonics and the observed fundamentals.

**Figure 3:**
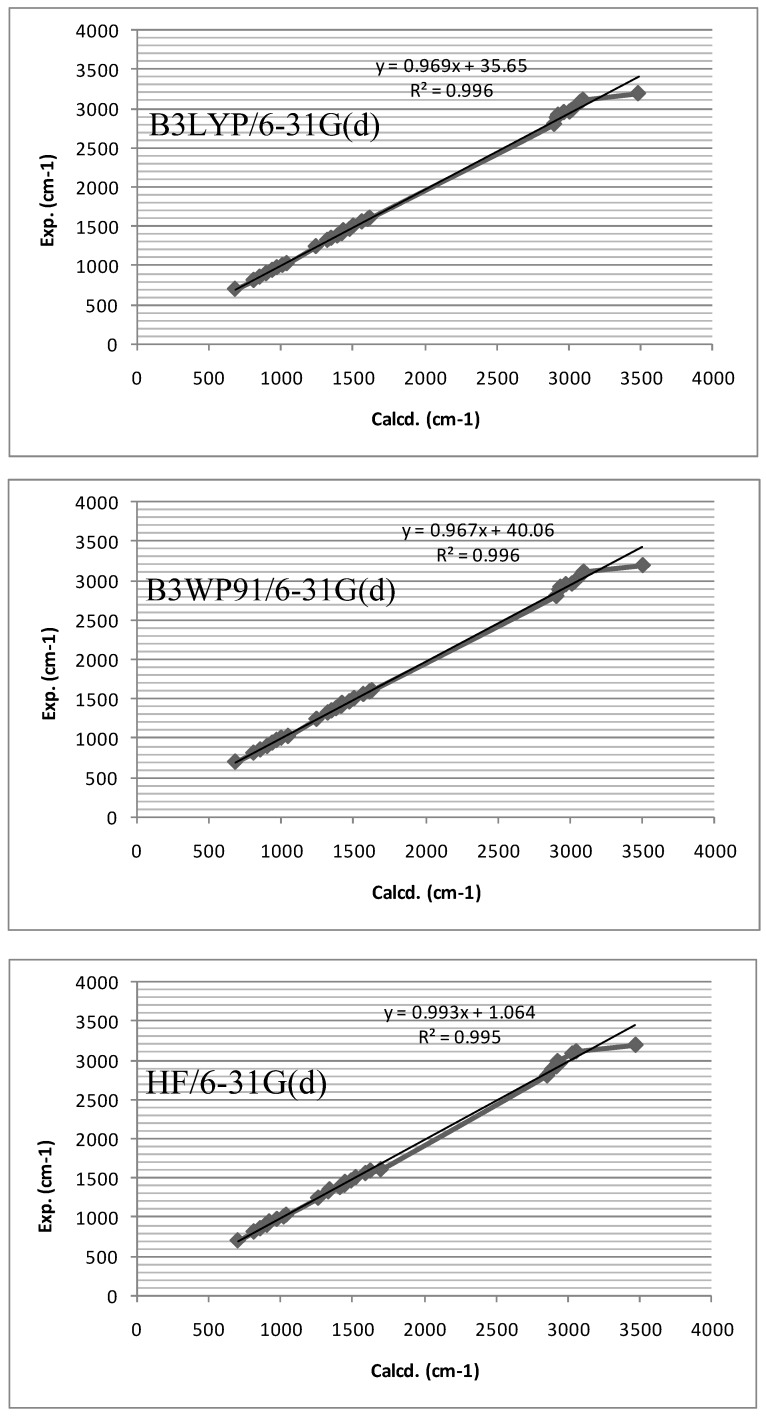
Correlations between the experimental and theoretical frequencies (cm^-1^) obtained by the B3LYP, B3WP91 and HF/6-31G(p) methods.

The resulting wave numbers for the optimized geometry and the experimental wave numbers together with the proposed assignments for compound **2** are given in Table 2. The vibrational spectral data obtained from the solid-phase FT-IR spectra based on the results of the normal coordinates calculations. The observed and the calculated spectra reflect a reasonable agreement for the vibrational frequencies. Based on the comparison of the calculated and experimental results, assignments of fundamental frequencies incorporate the observed band frequencies in the infrared spectra of the studied species confirmed by establishing a one-to-one correlation between observed and theoretically calculated frequencies. The calculated frequencies are slightly higher than the observed values for the majority of the normal modes. Many different factors may be responsible for the discrepancies between the experimental and computed spectra of the compound. Factors such as environment, anharmonicity, Fermi resonance, solvent effects and so forth are usually not considered in computations. The vibrational analysis is summarized in Table 2. Assignments of all vibrational bands has been carried out on the basis of mthe B3LYP calculations. A linearity between the experimental and the calculated wave numbers can be estimated by plotting the calculated vs. experimental wave numbers (Figure 3). The values of the correlation coefficients, 0.995-0.996, provide good linearity between the calculated and the experimental wave numbers. Better correlation had been established by B3LYP method. 

**Table 2:** Calculated Vibrational Frequencies (cm^-1^) of the most stable tautomer and the observed frequencies of compound **2**.

B3LYP	B3WP91	HF	Exp.	Assignment
3481	3498	3470	3200	υ(NH)
3094	3088	3055	3117	υ(CH)_C=C-H_
3081	3079	3030	3090	υ(CH)_phenyl_
3022	3025	2955	2991	υ(CH)
3004	3005	2940	2966	υ(CH)
2965	2964	2922	2958	υ(CH)
2926	2930	2917	2925	υ(CH)
2920	2921	2890	2891	υ(CH)
2900	2901	2851	2808	υ(CH)
1616	1924	1693	1600	υ(C=N)
1605	1609	1618	1593	υ(C=C)+ (C=N)
1561	1566	1577	1560	υ(C=C)
1503	1502	1518	1508	δ(N-H)
1478	1470	1486	1466	δ(Me)
1432	1421	1443	1447	δ(CH_2_)
1426	1414	1441	1410	ω(Me)_N-Me_
1392	1381	1411	1381	ω (Me)_C-Me_
1351	1346	1336	1368	δ(CH_2_)
1321	1322	1330	1297	δ(C-H)_C=C-H_
1241	1256	1256	1241	υ(CH_2_-C-CH_2_) _asym._
1037	1047	1032	1027	υ(Me)_N-Me_
1011	1000	1017	1005	Ph breath
974	969	972	975	Ph breath
940	938	916	926	δ_ring_
895	902	901	896	υ(CH_2_-C-CH_2_)_sym_
851	857	853	840	υ(C-C )
811	810	811	787	δ_ring_
683	681	700	705	δ_ring_

## Experimental

### General

Melting points were measured on a Buchi melting point apparatus B-510 (Buchi Labortechnik AG, Switzerland) and are uncorrected. Micro analytical data were obtained with a Vario Elemental apparatus (Shimadzu, Japan). NMR spectra were recorded at 400 MHz (^1^H) or 100 MHz (^13^C) using a Jeol 400 GX JNM spectrometer (Japan) with TMS as internal standard. Data are reported in ppm on the δ scale. IR spectra were recorded as KBr discs on a Shimadzu FT/IR spectrometer. Enaminothiones were prepared according to previously described procedure [12].

### General method for the preparation of diimines

Appropriate amounts of the enaminothione and the amine (see individual entries below) were dissolved in chloroform (30 mL) and the mixture was refluxed for 4 h. At the reaction completion as indicated by TLC the reaction mixture was cooled to room temperature and hexane was added to induce precipitation of the product which was then recrystallized from ethanol.

*5,5-dimethyl-3-benzylimino-cyclohex-1-enyl-benzylamine* (**1**): White powder. From **Ia** (1g, 4.08 mmol) and benzylamine (0.44g, 4.08 mmol). Yield 0.68 g (53%); mp 228-230°C; ^1^H-NMR (CDCl_3_): δ 0.89 (s, 6H, 2CH_3_), 2.43 (s, 2H, CH_2_), 2.47 (s, 2H, CH_2_), 4.24 (d, 2H, *CH_2_*-Ph), 4.36 (d, 2H, C*H_2_*-Ph), 4.86 (s, 1H, C=*CH*), 7.16 (m, 10H, Ar-*H*), 10.29 (s, 1H, NH); ^13^C-NMR (CDCl_3_) : δ 27.4, 32.4, 42.7, 46.7, 85.2, 94.8, 127.0, 127.6, 128.7, 135.4, 167.0, 169.4; Anal. Calcd. for C_22_H_26_N_2_: C, 82.97; H, 8.22; N, 8.79. Found: C, 82.69; H, 8.1; N, 8.58; IR (KBr, cm^-1^); 3175, 3125, 3025, 29752958, 2866, 1605, 1589, 1568, 1541, 1452, 1383, 1322, 1300, 1242, 1166, 1063, 992, 941, 886, 815, 763, 702, 668.

*5,5-dimethyl-3-propylimino-cyclohex-1-enyl-phenylamine* (**2**). White powder. From **Ib** (1 g, 4.08 mmol) and propylamine (0.26 g, 4.32 mmol). Yield 0.69 g (62%); mp 193-195° C; ^1^H-NMR (CDCl_3_): δ 0.86 (t, 3H, CH_3_), 0.93 (s, 6H, 2CH_3_), 1.63 (m, 2H, *CH_2_*-CH_3_), 2.54 (s, 2H, CH_2_), 2.77 (s, 2H, CH_2_), 3.04 (t, 2H, *CH_2_*CH_2_), 5.45 (s, 1H, *CH*=C), 7.28 (m, 5H, Ar-*H*), 9.97 (s, NH), 11.27 (s, NH); ^13^C-NMR (CDCl_3_):δ 11.5, 21.1, 27.5, 32.2, 42.7, 45.1, 84.8, 124.7, 126.9, 127.6, 129.4, 137.2, 168.0, 170.6; Anal. Calcd. for C_17_H_24_N_2_: C, 79.63; H, 9.45; N, 10.92. Found: C, 79.38; H, 9.38; N, 10.71; IR (KBr, cm^-1^): 3200, 3117, 3090, 2991, 2966, 2958, 2925, 2891, 2808, 1605, 1593, 1560, 1508, 1466, 1447, 1410, 1381, 1368, 1297, 1241, 1027, 1005, 975, 926, 896, 840, 787, 705.

*5,5-dimethyl-3-phynylimino-cyclohex-1-enyl-(4-methylphenylamine)* (**3**). White powder. From **Ic** (1 g, 4.1 mmol) and aniline (0.38 g, 4.1 mmol). Yield 0.59 g (45%); mp 237°C(dec); ^1^H-NMR (d_6_-DMSO): δ 1.14 (s, 6H, 2CH_3_), 2.28 (s, 3H, CH_3_), 2.49 (s, 2H, CH_2_), 2.62 (s, 2H, CH_2_), 5.78 (s, 1H, *CH*=C), 7.33 (m, 9H, Ar-*H*), 11.26 (s, 1H, NH); Anal. Calcd. for C_21_H_24_N_2_: C, 82.63; H, 7.94; N, 9.20. Found: C, 82.63; H, 7.82; N, 9.00; IR (KBr, cm^-1^): 3158, 3117, 3050, 2967, 2916, 2900, 2833, 2800, 1601, 1560, 1548, 1462, 1380, 1290, 1215, 1155, 1065, 1027, 937, 885, 825, 768, 701.

*5,5-dimethyl-3-phenylimino-cyclohex-1-enyl-phenylamine* (**4**). White powder. From **Ib** (1 g, 4.30 mmol) and aniline (0.4 g, 4.30mmol). Yield 0.72 g (57%); mp 290° C (dec); ^1^H-NMR (d_6_-DMSO): δ 1.07 (s, 6H, 2CH_3_), 2.49 (s, 2H, CH_2_), 2.65 (s, 2H, CH_2_), 5.80 (s, 1H, *CH*=C), 7.34 (m, 10, Ar-*H*), 11.38 (s, 1H, NH); Anal. Calcd. for C_20_H_22_N_2_: C, 82.71; H, 7.63; N, 9.64. Found: C, 82.40; H, 7.42; N, 9.41; IR (KBr, cm^-1^): 3167, 3117, 3051, 2983, 2966, 2866, 2816, 1601, 1560, 1545, 1451, 1372, 1282, 1211, 1151, 1057, 997, 941, 862, 802, 765, 708, 697.

*5,5-dimethyl-3-phenylamino-cyclohex-1–enyl-(4-chlorophenylamine)* (**5**)***.*** Faint yellow powder. From **Id** (1 g, 4.10 mmol) and aniline (0.37 g, 4.00 mmol). Yield: 0.5 g (38%); mp 300° C (dec);^1^H-NMR (d_6_-DMSO): δ 0.98 (s, 6H, 2CH_3_), 2.48 (s, 2H, CH_2_), 2.64 (s, 2H, CH_2_), 5.81 (s, 1H, *CH*=C), 7.41 (m, 9H, Ar-*H*), 11.5 (s, 1H, NH); Anal. Calcd. for C_20_H_21_N_2_: C, 73.94; H, 6.51; N, 8.62. Found: C, 73.69; H, 6.43; N, 8.41; IR (KBr, cm^-1^): 3167, 3117, 3033, 2958, 2916, 2866, 2800, 1599, 1561, 1551, 1452, 1397, 1289, 1210, 1152, 1063, 1002, 941, 879, 760, 709, 669.

*5,5-dimethyl-3-benzylimino-cyclohex-1-enyl-phenylamine* (**6**). White powder. From **Ia** (1 g, 4.1 mmol) and aniline (0.38 g, 4.1 mmol). Yield: 0.53 g (40%); mp 203-205°C; ^1^H-NMR (CDCl_3_): δ 0.97 (s, 6H, 2CH_3_), 2.55 (s, 2H, CH_2_), 2.64 (s, 2H, CH_2_), 4.28 (d, 2H, *CH*-Ph), 5.34 (s, 1H, *CH*=C), 7.11 (m, 10H, Ar-*H*), 10.50 (s, NH), 11.25 (s, NH); Anal. Calcd. for C_21_H_24_N_2_: C, 82.85; H, 7.94; N, 9.20. Found: C, 82.62; H, 7.89; N, 8.91; IR (KBr, cm^-1^): 3201, 3173, 3125, 3058, 2971, 2951, 2904, 2846, 2807, 1605, 1538, 1452, 1384, 1307, 1260, 1163, 1000, 923, 888, 808, 779, 663.

### Computational method

The studied structures were fully geometry optimized using PCGAMESS [13] computational program for calculations. The calculations done at the B3LYP/6-31G(d,p) as well as B3LP, B3WP91 and HF levels using the basis set 6-31G(d). Vibrational frequencies were calculated using the last three levels [11].

## Conclusions

In this study both symmetrical and non-symmetrical diimines of dimedone were synthesized by direct reaction of their corresponding enaminothiones with primary amines. Non-symmetrical substitution could be established by varying both the substituent on the nitrogen atom of the enaminothione and the amine.
